# Changes in Serum GDF-15 in Relation to the Onset of Post-Stroke Depression After Acute Ischemic Stroke and the Development of a Comprehensive Prediction Model

**DOI:** 10.62641/aep.v54i4.2230

**Published:** 2026-08-15

**Authors:** Chengzhi Guo, Xiuyan Liu, Yu Yun, Zhihuan Mi

**Affiliations:** ^1^Research and Development Department (R&D), Beijing Lihe Innotech Co., Ltd., 100023 Beijing, China; ^2^Department of Clinical laboratory, Siyang County Hospital of Traditional Chinese Medicine, 223700 Suqian, Jiangsu, China; ^3^Neurology Department, Siyang County Hospital of Traditional Chinese Medicine, 223700 Suqian, Jiangsu, China; ^4^Pediatric and Adolescent Ward, Kailuan Mental Health Center, 063000 Tangshan, Hebei, China

**Keywords:** growth differentiation factor-15, Ischemic stroke, post-stroke depression, nomogram, risk factors

## Abstract

**Background:**

This study aimed to investigate the relationship between serum growth differentiation factor-15 (GDF-15) levels and the development of post-stroke depression (PSD) following acute ischemic stroke (AIS) and to construct an integrated predictive model.

**Methods:**

This retrospective study enrolled 302 patients with AIS admitted to Siyang County Hospital of Traditional Chinese Medicine between January 2023 to October 2025. Among them, 100 patients developed PSD, and the remaining 202 patients did not. Serum GDF-15 levels at admission, general clinical data and disease-related information were compared between groups. Logistic regression identified independent risk factors for PSD, and a nomogram-based prediction model was developed. Model performance was assessed using receiver operating characteristic (ROC) analysis.

**Results:**

Compared with the non-PSD group, the PSD group had older age, higher National Institutes of Health Stroke Scale (NIHSS) scores, and a greater proportion of patient with a family history of psychiatric disorders and frontal lobe infarction (*p* < 0.05). Serum level of high-sensitivity C-reactive protein (hs-CRP), homocysteine, uric acid, Interleukin-6 (IL-6), neurofilament light chain (NfL) and GDF-15 were elevated, whereas left ventricular ejection fraction was lower in the PSD group (*p* < 0.05). Logistic regression showed that higher NIHSS scores (odds ratio (OR) = 1.230, 95% confidence interval (CI): 1.045–1.447), family history of psychiatric disorders (OR = 7.760, 95% CI: 1.709–35.238), frontal lobe infarction (OR = 5.275, 95% CI: 1.574–17.678), and elevated level of IL-6 levels (OR = 1.319, 95% CI: 1.089–1.599), NfL (OR = 1.103, 95% CI: 1.018–1.195), and GDF-15 (OR = 1.021, 95% CI: 1.009–1.033) were independent risk factors for PSD in patients with AIS (*p* < 0.05). The nomogram achieved an area under the ROC curve of 0.863 (95% CI: 0.810–0.885) for predicting PSD in patients with AIS.

**Conclusions:**

Serum GDF-15 is closely associated with PSD onset after AIS. A comprehensive prediction model integrating GDF-15 with other clinical factors demonstrates high clinical utility for predicting PSD risk.

## Introduction

Acute ischemic stroke (AIS) is a common cerebrovascular disorder associated with high disability and recurrence rate. Although thrombolytic and endovascular therapies have improved survival, post-stroke neuropsychiatric complications continue to severely impact patient prognosis and quality of life [1]. Among these complications, post-stroke depression (PSD) is one of the most prevalent psychological sequelae of AIS. PSD not only reduces rehabilitation adherence and hinders neurological recovery but also increases the risk of cognitive impairment and mortality [2].

The pathogenesis of PSD is complex, involving neurobiological, psychosocial, and environmental factors. Furthermore, early clinical symptoms of PSD frequently overlap with stroke-related neurological deficits, leading to underrecognition or misdiagnosis in clinical practice [3]. Therefore, identifying objective and sensitive biomarkers for the early detection of individuals at high risk for PSD is crucial for developing individualized intervention strategies.

In recent years, the role of neuroinflammation and oxidative stress in PSD pathogenesis has gained increasing attention. Growth differentiation factor-15 (GDF-15), a member of the transforming growth factor-beta superfamily, is a classic stress-response protein [4]. Under pathological conditions such as ischemia-hypoxia, inflammation, and tissue injury, GDF-15 expression is markedly upregulated, where it regulates inflammatory responses and apoptotic processes [5, 6]. Existing evidence indicates that GDF-15 is closely associated with cardiovascular diseases and various neurodegenerative disorders [7]. However, its role in post-stroke affective disorders remains relatively understudied, and its clinical applicability warrants further exploration. This study aimed to investigate the relationship between serum GDF-15 levels and PSD occurrence in patients with AIS. Furthermore, by integrating clinical characteristics and other serological markers, we constructed a nomogram-based comprehensive prediction model. This endeavour seeks to provide a scientific basis for the early clinical screening, prevention and treatment of PSD.

## Materials and Methods

### General Data

This retrospective study extracted data from the hospital’s medical record system. The sample size was determined based on the rule of thumb requiring at least 10–15 events per variable for multivariate logistic regression analysis [8]. Based on the literature, approximately 6–10 potential influencing factors were identified from literature; thus, a minimum of 60–100 patients with PSD were required [8]. Given the reported PSD incidence of 30%–50% [9], we initially screened 302 patients with AIS to ensure at least 100 PSD cases for stable model estimation. To minimize bias from sample size in the real-world data collection, this study enrolled 302 patients with AIS admitted to Siyang County Hospital of Traditional Chinese Medicine between January 2023 and October 2025. Among them, 100 patients diagnosed with PSD constituted the PSD group, and the remaining 202 without PSD served as the non-PSD group. All participants provided informed consent. The study was approved by the Ethics Committee of Siyang County Hospital of Traditional Chinese Medicine (Approval No.: SYZYIRB-2026-023) and complied the relevant requirements of the Declaration of Helsinki for medical research.

The diagnostic criteria were as follows: (1) AIS was diagnosed based on the criteria outlined in the Chinese Guidelines for the Diagnosis and Treatment of Acute Ischemic Stroke 2018 [10]; and (2) PSD was diagnosed according to the diagnostic criteria for depressive episodes specified in either the “International Classification of Diseases, 11th Revision (ICD-11)” [11] or the “Diagnostic and Statistical Manual of Mental Disorders, Fifth Edition (DSM-5)” [12]. Patient presented with either a substantial and persistent depressed mood or a marked reduction in interest or pleasure in all or almost all activities. A psychiatrist at the associate chief physician level or above made the diagnosis within 2 to 4 weeks after the stroke, with symptoms lasting for at least 2 weeks.

The inclusion criteria were as follows: (1) Aged ≥50 years; (2) presence of ischemic lesions on cranial CT and MRI examinations with a definitive diagnosis; (3) first-ever ischemic stroke documented in medical records; and (4) Glasgow Coma Scale score of ≥13 upon admission and ability to cooperate with the investigation, as documented in medical records [13].

The exclusion criteria were as follows: (1) History of intracranial tumour or traumatic brain injury; (2) history of psychiatric illness or antipsychotic medication use; (3) concurrent or previous haemorrhagic stroke; or (4) concurrent severe systemic diseases, such as advanced cancer, liver and kidney failure, or other neurological disorders.

### Collection of Baseline Data

Using a predefined data extraction form, researchers uniformly extracted and verified patient general data from the hospital’s medical record system. These data included age, gender, body mass index, years of education, time from onset to admission, smoking status, alcohol consumption, family income, and information on underlying diseases such as hypertension, diabetes, hyperlipidaemia, coronary heart disease, and chronic pulmonary disease. Underlying diseases were identified based on documented definitive diagnoses and long-term medication records. Family history of psychiatric illness was determined using information recorded in the admission face sheet, initial progress note, admission note, and previous visit records. Admission stroke severity was extracted based on the National Institutes of Health Stroke Scale (NIHSS) [14] score recorded by neurologists in the medical records. The NIHSS assesses consciousness and orientation, visual function, motor function, sensation and coordination, language function, and neglect, with a total score ranging from 0 to 42; higher scores indicate more severe neurological deficits. Infarct location was determined based on cranial MRI (preferred) or CT imaging reports and was classified as frontal lobe, basal ganglia, brainstem, cerebellum, and other regions [15, 16]. Left ventricular ejection fraction (LVEF) was extracted from echocardiography results documented during hospitalization.

### Collection of Biochemical Indicators

Biochemical and inflammation-related indicators were extracted from routine laboratory records obtained after admission. For all laboratory parameters, venous blood test results recorded within 24 hours after admission, primarily from morning fasting samples, were selected. Serum albumin (ALB), total bilirubin (TBIL), uric acid (UA), homocysteine (Hcy) and high-sensitivity C-reactive protein (hs-CRP) were extracted from test results completed by the clinical laboratory according to standard procedures and subjected to internal quality control. Routine blood parameters, including white blood cell count (WBC), neutrophil count (NEU), lymphocyte count (LYMP), haemoglobin (Hb) and platelet count (PLT), were extracted from the same-period haematology analysis results. Interleukin-6 (IL-6), neurofilament light chain (NfL) and GDF-15 were extracted from test records completed by the clinical laboratory using uniform methods, the relevant results and units were as documented in the medical record system.

### Statistical Processing

All data were analysed using R software (version 4.0.0; R Core Team, Vienna, Austria). Continuous variables were assessed for normality using the Kolmogorov–Smirnov test and met the assumptions of normal distribution. They are expressed as mean ± standard deviation and were compared between groups using independent samples *t*-test. Categorical variables are described as frequencies and percentages (%), and group comparisons were performed using the chi-square test. Multicollinearity among independent variables was evaluated using the variance inflation factor (VIF). Multivariate analysis of factors associated with PSD was performed using logistic regression. A nomogram was constructed for the prediction model. Internal validation was conducted using bootstrap resampling. Model performance was evaluated using the receiver operating characteristic (ROC) curve. Missing data were handled by listwise deletion. A *p*-value < 0.05 was considered statistically significant.

## Results

### Comparison of Baseline Characteristics Between the PSD Group and the Non-PSD Group

The PSD group had significantly older age and higher NIHSS score than the non-PSD group. The proportion of patients with a family history of psychiatric disorders and with frontal lobe infarction were also significantly greater in the PSD group (*p* < 0.05). No significant differences (*p* > 0.05) were observed between the two groups in body mass index, years of education, time from onset to admission, gender distribution, smoking status, alcohol consumption, prevalence of hypertension, diabetes, hyperlipidaemia, coronary heart disease, chronic pulmonary disease, or family income (Table 1).

**Table 1.** **Comparison of baseline characteristics between the PSD group and the non-PSD group**.

Indicators		PSD group (n = 100)	non-PSD group (n = 202)	*t*	χ^2^	*p*
Age (years)		68.8 ± 7.6	66.4 ± 8.3	2.431		0.016
BMI (kg/m^2^)		23.78 ± 1.74	23.86 ± 1.93	–0.350		0.727
NIHSS score (point)		4.63 ± 1.00	3.22 ± 0.81	13.145		<0.001
Years of education		5.86 ± 1.94	5.98 ± 1.97	–0.501		0.617
Time from onset to admission (hours)		13.76 ± 3.55	14.22 ± 3.87	–0.999		0.319
Gender (%)					0.976	0.323
	Female	42(42.00)	97(48.02)			
	Male	58(58.00)	105(51.98)			
Stroke location (n [%])					12.455	0.014
	Frontal lobe	32(32.00)	31(15.35)			
	Basal ganglia	40(40.00)	98(48.51)			
	Brainstem	13(13.00)	32(15.84)			
	Cerebellum	8(8.00)	15(7.43)			
	Other	7(7.00)	26(12.87)			
Smoking status (n [%])					0.115	0.734
	Yes	42(42.00)	89(44.06)			
	No	58(58.00)	113(55.94)			
Alcohol consumption (n [%])					2.088	0.148
	Yes	31(31.00)	47(23.27)			
	No	69(69.00)	155(76.73)			
Type of comorbid underlying diseases (n [%])						
	Hypertension	69(69.00)	121(59.9)		2.373	0.123
	Diabetes	24(24.00)	58(28.71)		0.751	0.386
	Hyperlipidaemia	74(74.00)	148(73.27)		0.018	0.892
	Coronary heart disease	10(10.00)	27(13.37)		0.705	0.401
	Chronic pulmonary disease	16(16.00)	41(20.30)		0.876	0.319
Family income (n [%])					3.541	0.060
	≤6000 CNY	76(76.00)	132(65.35)			
	>6000 CNY	24(24.00)	70(34.65)			
Family history of psychiatric disorders (n [%])					18.712	<0.001
	Yes	17(17.00)	6(2.97)			
	No	83(83.00)	196(97.03)			

Note: PSD, post-stroke depression; BMI, body mass index; NIHSS, National Institutes of Health Stroke Scale; CNY, Chinese Yuan. 1 CNY ≈ 0.1394 USD.

### Comparison of Various Indicators Between the PSD Group and the Non-PSD Group

Serum levels of hs-CRP, Hcy, UA, IL-6, NfL, and GDF-15 were significantly higher in the PSD group than in the non-PSD group, whereas LVEF was significantly lower in the PSD group (*p* < 0.05). In contrast, no significant differences (*p* > 0.05) were observed between the two groups in ALB, PLT, Hb, NEU, LYMP, and TBIL (Table 2).

**Table 2.** **Comparison of various indicators between the PSD group and the non-PSD group**.

Indicators	PSD group (n = 100)	non-PSD group (n = 202)	*t*	*p*
ALB (g/L)	40.14 ± 3.10	40.20 ± 2.97	–0.163	0.871
PLT (× 109/L)	228.2 ± 20.0	224.6 ± 22.1	1.374	0.170
WBC (× 109/L)	9.8 ± 1.5	10.0 ± 2.0	–0.884	0.377
NEU (× 109/L)	5.6 ± 0.8	5.8 ± 1.0	–1.742	0.082
LYMP (× 109/L)	2.05 ± 0.42	2.11 ± 0.46	–1.097	0.273
Hb (g/L)	132.7 ± 7.1	133.5 ± 7.8	–0.864	0.388
hs-CRP (mg/L)	15.11 ± 2.79	12.87 ± 2.63	6.826	<0.001
LVEF (%)	41.74 ± 2.62	42.65 ± 2.95	–2.616	0.009
Hcy (μmol/L)	18.03 ± 2.59	15.32 ± 2.33	9.163	<0.001
TBIL (μmol/L)	16.49 ± 3.14	15.93 ± 3.76	1.284	0.200
UA (μmol/L)	319.00 ± 37.98	305.19 ± 36.65	3.045	0.003
IL-6 (pg/mL)	12.06 ± 1.81	8.33 ± 1.72	17.430	<0.001
NfL (pg/mL)	76.58 ± 9.18	54.57 ± 6.52	23.992	<0.001
GDF-15 (ng/L)	1858.10 ± 202.15	1567.89 ± 201.71	11.758	<0.001

Note: PSD, post-stroke depression; ALB, albumin; PLT, platelet count; WBC, white blood cell count; NEU, neutrophil count; LYMP, lymphocyte count; Hb, hemoglobin; hs-CRP, high-sensitivity C-reactive protein; LVEF, left ventricular ejection fraction; Hcy, homocysteine; TBIL, total bilirubin; UA, uric acid; IL-6, interleukin-6; NfL, neurofilament light chain; GDF-15, growth differentiation factor-15.

### Multivariate Analysis of PSD Onset

Variables that were significant in the univariate analysis—including age, NIHSS score, presence of a family history of psychiatric disorders, frontal lobe infarction, hs-CRP, Hcy, UA, IL-6, NfL, GDF-15 levels, and LVEF—were included as independent variables. Firstly, VIF was calculated to assess multicollinearity. After excluding UA and hs-CRP, all remaining independent variables had VIF values ≤5, indicating well-controlled multicollinearity. Logistic regression revealed that the following were independent risk factors for PSD in patients with AIS (all *p* < 0.05): higher NIHSS score (odds ratio (OR) = 1.230, 95% confidence interval (CI): 1.045–1.447), family history of psychiatric disorders (OR = 7.760, 95% CI: 1.709–35.238), frontal lobe infarction (OR = 5.275, 95% CI: 1.574–17.678), higher level of IL-6 (OR = 1.319, 95% CI: 1.089–1.599), NfL (OR = 1.103, 95% CI: 1.018–1.195), and GDF-15 (OR = 1.021, 95% CI: 1.009–1.033, Table 3).

**Table 3.** **Multivariate analysis of PSD onset**.

Indicators		β	SE	Wald χ^2^	*p*	OR	95% CI
Age		0.014	0.008	3.063	0.089	1.014	0.998–1.030
NIHSS score		0.207	0.083	6.220	<0.001	1.230	1.045–1.447
Lesion location							
	Basal ganglia					1.000	
	Brainstem	0.102	0.08	1.626	0.322	1.107	0.947–1.295
	Cerebellum	0.092	0.07	1.727	0.313	1.096	0.956–1.258
	Other	0.304	0.192	2.507	0.251	1.355	0.930–1.975
Frontal lobe		1.663	0.617	7.265	<0.001	5.275	1.574–17.678
Family history of psychiatric disorders		2.049	0.772	7.044	<0.001	7.760	1.709–35.238
LVEF		–0.133	0.113	1.385	0.384	0.875	0.702–1.093
Hcy		0.304	0.189	2.587	0.227	1.355	0.936–1.963
IL-6		0.277	0.098	7.989	<0.001	1.319	1.089–1.599
NfL		0.098	0.041	5.713	0.003	1.103	1.018–1.195
GDF-15		0.021	0.006	12.250	<0.001	1.021	1.009–1.033
Constant		1.639	0.788	4.326	0.046	-	-

Note: SE, standard error; OR, odds ratio; CI, confidence interval; PSD, post-stroke depression; NIHSS, National Institutes of Health Stroke Scale; LVEF, left ventricular ejection fraction; Hcy, homocysteine; IL-6, interleukin-6; NfL, neurofilament light chain; GDF-15, growth differentiation factor-15.

### Construction and Validation of the Predictive Model for PSD Onset

**Fig. 1.**
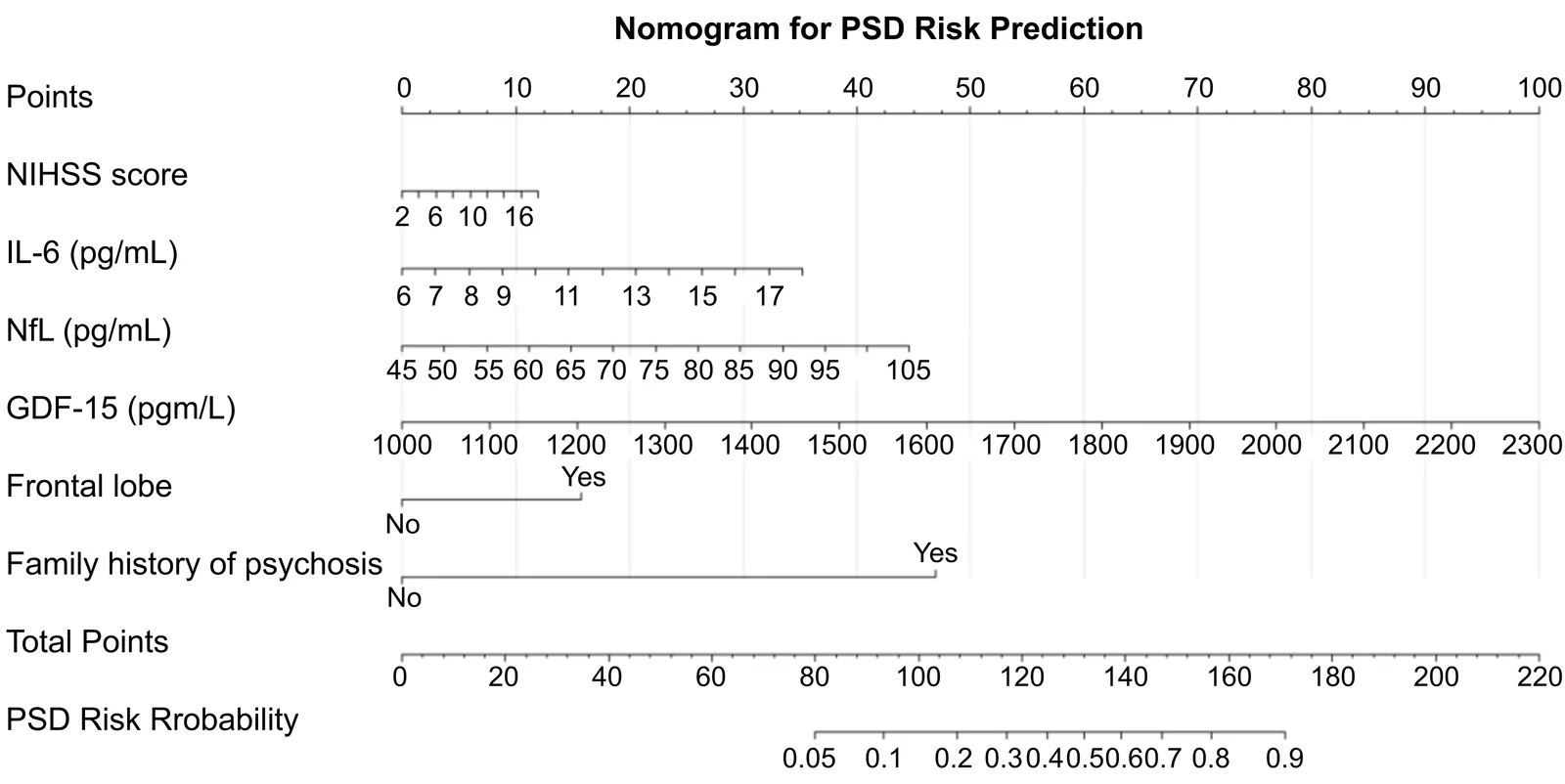
**Nomogram prediction model for PSD onset**. Note: PSD, post-stroke depression; NIHSS, National Institutes of Health Stroke Scale; IL-6, interleukin-6; NfL, neurofilament light chain; GDF-15, growth differentiation factor-15.

**Fig. 2.**
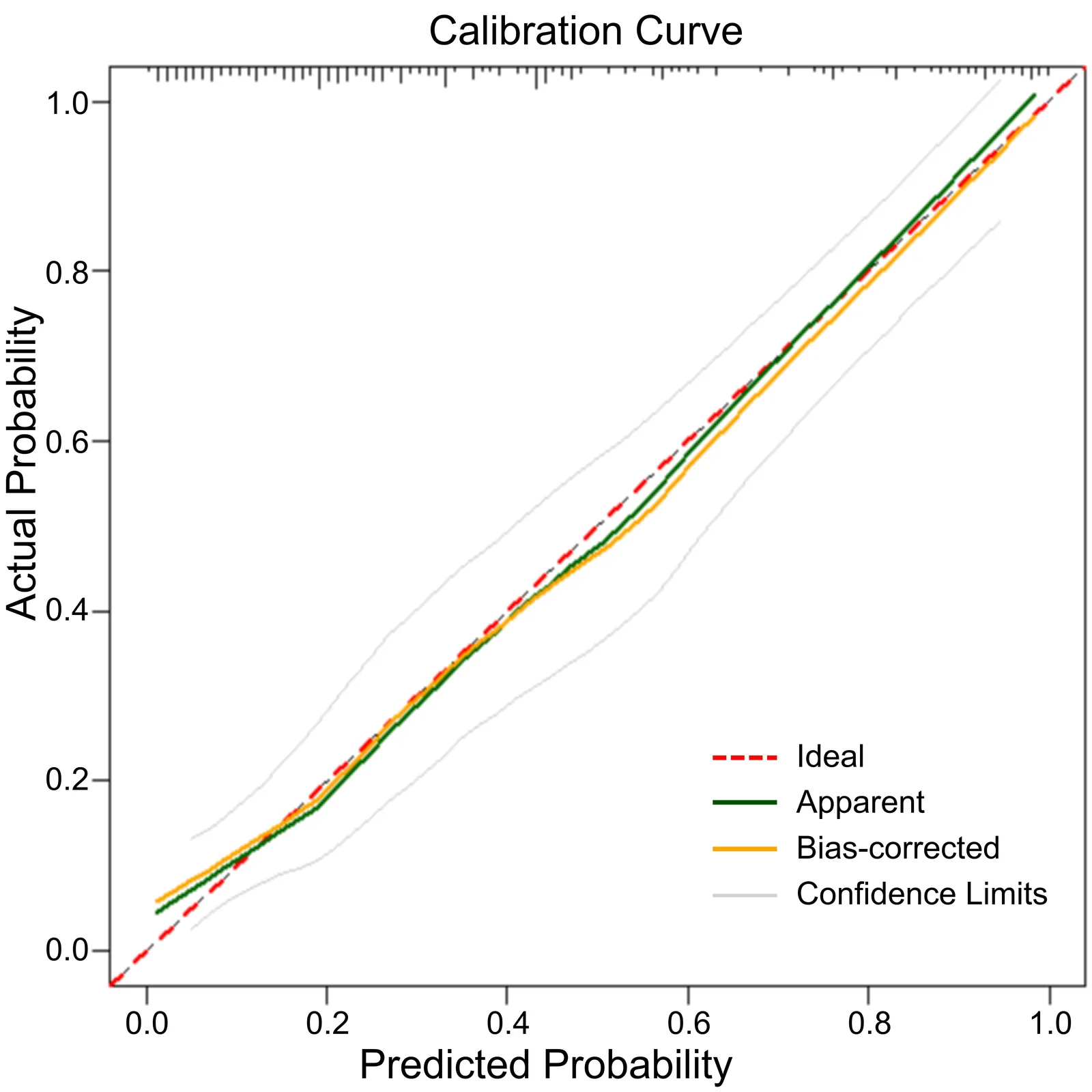
**Calibration curve for internal validation**.

**Fig. 3.**
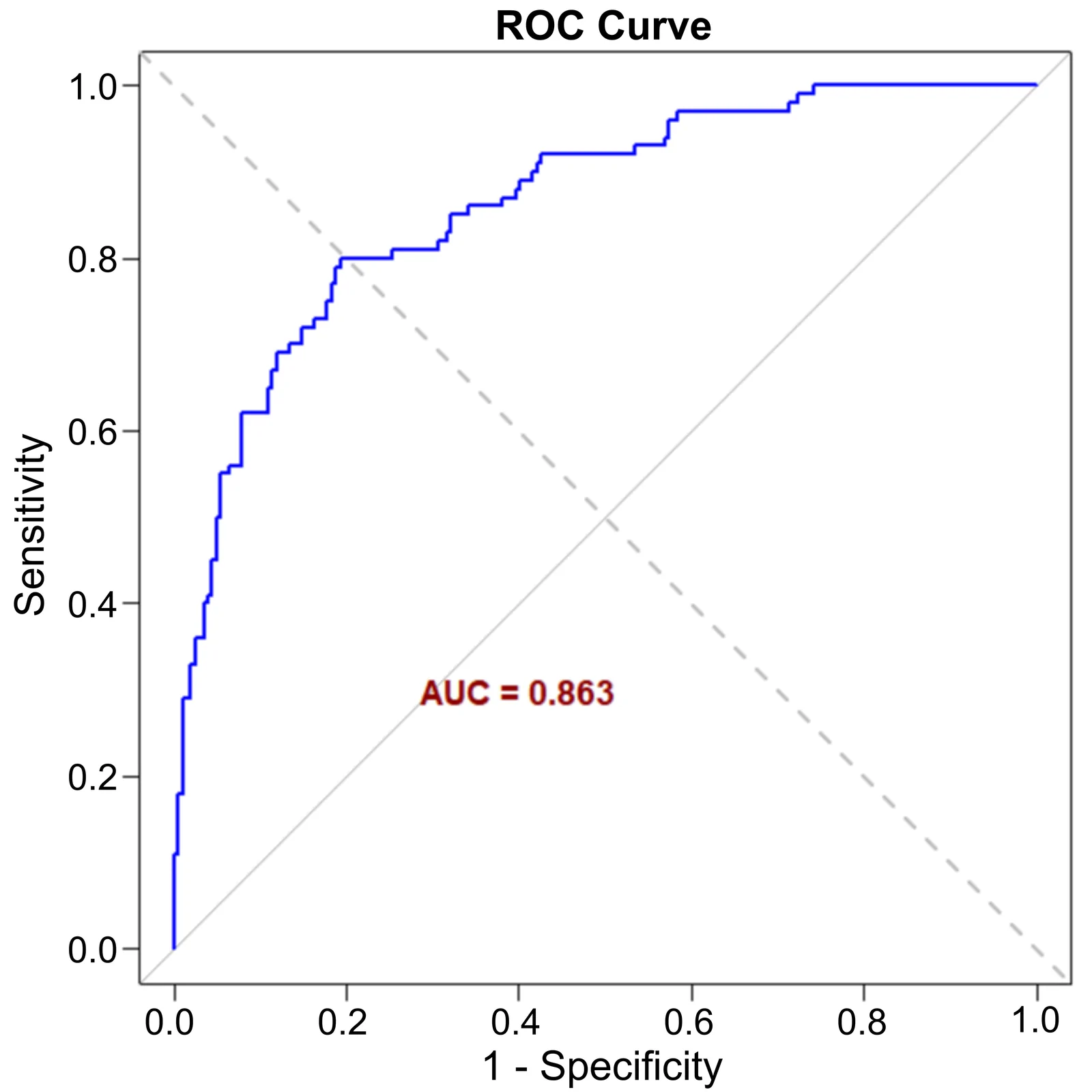
**ROC curve of the nomogram for predicting PSD in patients with acute ischemic stroke**. Note: PSD, post-stroke depression; ROC, receiver operating characteristic; AUC, area under the ROC curve.

A nomogram prediction model was constructed based on the independent variables included in the logical regression model. The scores corresponding to different values of NIHSS score, family history of psychiatric disorders, frontal lobe infarction, and levels of IL-6, NfL, and GDF-15 are shown in Fig. 1. The total score ranges from 80 to 170 points. The predicted probability of PSD corresponding to each total scores is indicated on the total point axis (bottom scale). A total score of 80 points the corresponds to a predicted PSD probability is 0.05, and a total score of 170 corresponds to a predicted probability of 0.90. A higher total score indicates greater PSD risk. Model goodness-of-fit was internally validated using bootstrap resampling, and a calibration curve was plotted (Fig. 2). The calibration curve showed high agreement among the ideal, apparent, and bias-corrected curve. The Hosmer–Lemeshow test yielded χ^2^ = 2.550, *p* = 0.107 (>0.05). The predictive value of the nomogram was evaluated using the ROC curve (Fig. 3). The area under the ROC curve (AUC) for predicting PSD in patients with AIS was 0.863 (95% CI: 0.810–0.885).

## Discussion

Accurately identifying PSD in clinical settings is challenging because its symptoms overlap with neurological deficits. Relying solely on scale assessments or patients’ subjective reports can lead to underdiagnosis and delayed intervention [17, 18]. GDF-15, a stress-responsive cytokine, reflects inflammation intensity and oxidative stress and is associated with adverse outcomes after cardiovascular and cerebrovascular events. Incorporating GDF-15 into PSD risk assessment may capture critical aspects of the body’s stress load during the acute stroke phase, the extent of neural tissue damage, and the involvement of mood-regulating pathways [19, 20].

In this study, the PSD group was older and had higher NIHSS scores than the non-PSD group, along with a higher prevalence of family history of psychiatric disorders and frontal lobe infarctions. Advancing age is often associated with a lower threshold for neuroinflammation, impaired mitochondrial homeostasis, and reduced cerebrovascular reserve. These factors collectively reduce the post-stroke compensatory capacity of neural networks, making the emotional regulation system more vulnerable to inflammatory and stress signals [21]. A higher NIHSS score can be considered a comprehensive indicator of both infarct burden and the degree of neurological dysfunction. Neurological deficits not only increase psychological stress related to disability but also suggest a stronger inflammatory cascade during the ischemia-reperfusion process. These factors together make patients more susceptible to developing depressive symptoms and progressing to PSD after a stroke [22]. Regarding stroke location, frontal lobe infarction was more common in the PSD group. The underlying mechanism may be that the frontal-limbic network is critically involved in emotion regulation, reward processing, and social cognition. Structural damage to the frontal lobe can disrupt the prefrontal-cingulate-amygdala regulatory circuitry, leading to a negative emotional bias and imbalance affective responses. Furthermore, altered coupling between the frontal lobe and networks such as the default mode and executive control networks may reduce emotion reappraisal capacity, allowing mild depressive symptoms to persist and consolidate into clinical PSD [23].

At the biomarker level, serum GDF-15 levels in the PSD group were higher than those in the control group. As a transforming growth factor-beta superfamily member, GDF-15 is not only an inflammation-related cytokine but also increasingly recognized as a sensitive signal of cellular mitochondrial stress [24]. Zang *et al*. [25] demonstrated in an AIS trial that the elevation of serum GDF-15 levels after AIS is linearly and positively correlated with PSD risk, consistent with our findings. Ischemia-hypoxia-triggered mitochondrial dysfunction can lead to compensatory GDF-15 overexpression. Literature suggests that GDF-15 may play a dual role as a damage marker and a neuroprotective factor in neurodegenerative diseases, and its elevation reflects the body’s intense stress response to ischemic injury and the inflammatory microenvironment [26]. Furthermore, Mastrobattista *et al*. [27] proposed that GDF-15 is a biomarker of accelerated aging and is closely associated with late-life depression and cognitive decline. Therefore, elevated serum GDF-15 may indicate that brain tissue in patients with PSD are in a state of accelerated immune aging, thereby weakening neuroplasticity and hindering the re-establishment of emotional homeostasis after an ischemic insult.

Our results also showed that hs-CRP, Hcy, UA, IL-6 and NfL level were higher in the PSD group than in the control group; however, IL-6, NfL, and GDF-15 were the factors ultimately retained in the multivariate analysis. These findings suggest that the associations of hs-CRP, Hcy, and UA with PSD largely reflect the common pathological background of inflammatory response, oxidative stress, and neural injury, whereas their independent effects diminish after including variables more central to the disease process. As an acute-phase inflammatory response indicator, hs-CRP exhibits strong parallel directionality with the inflammatory activation state reflected by IL-6, and its univariate effect may be partially absorbed by IL-6. Although UA is associated with enhanced oxidative stress in cerebral ischemia, it is also influenced by multiple factors including metabolic status, renal excretion, and inflammation; after controlling for collinearity, it did not enter the final model. Hcy, though elevated in the intergroup comparison, participates in PSD development mainly indirectly through endothelial injury, microcirculatory disturbances, and inflammatory amplification; its statistical association did not persist after adjustment for combined inflammatory and neural injury indicators. In contrast, NfL more directly reflects the extent of axonal structural damage and therefore retains independent predictive significance in the multivariate model [28, 29, 30].

Logistic regression showed that higher NIHSS score, family history of psychiatric illness, frontal lobe infarction, and higher levels of IL-6, NfL, and GDF-15 were risk factors for PSD in patients with AIS. Based on the VIF screening results, after excluding hs-CRP and UA, no significant collinearity was observed among the remaining variables, suggesting that although GDF-15, IL-6, and NfL all belong to inflammation-injury-related pathways, the pathological dimensions they represent are not completely overlapping. Specifically, GDF-15 is more strongly reflects systemic stress and mitochondrial homeostasis dysregulation, IL-6 mainly reflects the degree of inflammatory cascade activation, and NfL more directly indicates axonal structural damage. Therefore, including all three together remains complementary [31, 32, 33]. However, this study did not include interaction terms to analyse effect modification between GDF-15 and IL-6 or NfL; thus, whether their combined elevation exerts additive or amplifying effects requires clarification in future studies. Given the limited predictive value of any single indicator, this study constructed a comprehensive predictive nomogram incorporating clinical characteristics and biomarkers, which achieved an AUC of 0.863, indicating good predictive performance. Che *et al*. [34] confirmed that combining biomarkers from different pathological pathways improves risk stratification. Lu *et al*. [35] also found that incorporating GDF-15 into a traditional risk model increased the predictive efficacy for three months PSD to 0.88. The AUC is comparable to those previously reported, suggesting that GDF-15 has a relatively stable incremental value in PSD model construction. Compared with models that include only biological indicators, this study further integrated clinical variables such as family history of psychiatric illness and frontal lobe infarction, allowing the model to balance individual susceptibility and lesion anatomical characteristics, thereby enhancing clinical interpretability [36].

To ensure depression assessment reliability, this study excluded patients with severe aphasia, impaired consciousness, or inability to cooperate with the examination. This may have resulted in a study sample predominantly representing the subgroup of PSD patients with relatively preserved neurological function, thus, the findings may have limited applicability to patients with severe stroke. Furthermore, this study constructed a nomogram based on single-centre retrospective data, with relatively homogeneous sample sources, diagnostic and treatment workflows, and laboratory testing patterns; consequently, the model parameters may be influenced by the centre-specific case mix. In addition, we used only Bootstrap resampling for internal validation. Although the calibration curve and Hosmer–Lemeshow test indicated good model fit within this sample, this result primarily reflects internal stability and cannot replace external validation using an independent cohort. The discriminative ability, calibration, and generalizability of the model in different regions, hospital levels, and stroke populations have not yet been confirmed. Moreover, although we controlled for collinearity, we did not examine potential interactions between GDF-15 and IL-6 or NfL; therefore, our understanding of the combined effects of multiple pathway indicators remains limited. Future multicentre, prospective studies with larger sample sizes are needed to perform external validation and, together with dynamic follow-up data, further optimize the robustness and clinical applicability of the model.

## Conclusions

Serum GDF-15, a key molecule reflecting cellular stress and inflammatory status, is closely associated with PSD onset after AIS. The underlying mechanism may involve neuroimmune dysregulation mediated by mitochondrial dysfunction. The integrated predictive model, constructed by combining serum GDF-15, inflammatory cytokines, neuronal injury biomarkers, and clinical imaging features, captures PSD risk from multiple pathophysiological dimensions and demonstrates high potential for clinical translation and application value.

## Availability of Data and Materials

All experimental data included in this study can be obtained by contacting the corresponding author if needed.
